# The Relative Power of Structural Genomic Variation versus SNPs in Explaining the Quantitative Trait Growth in the Marine Teleost *Chrysophrys auratus*

**DOI:** 10.3390/genes13071129

**Published:** 2022-06-23

**Authors:** Mike Ruigrok, Bing Xue, Andrew Catanach, Mengjie Zhang, Linley Jesson, Marcus Davy, Maren Wellenreuther

**Affiliations:** 1 The New Zealand Institute for Plant & Food Research Ltd., Nelson 7010, New Zealand; mikeruigrok@live.nl (M.R.); andrew.catanach@plantandfood.co.nz (A.C.); linley.jesson@plantandfood.co.nz (L.J.); marcus.davy@plantandfood.co.nz (M.D.); 2 Wellington Faculty of Engineering, Victoria University of Wellington, Wellington 6012, New Zealand; bing.xue@vuw.ac.nz (B.X.); mengjie.zhang@vuw.ac.nz (M.Z.); 3 School of Biological Sciences, University of Auckland, Auckland 1010, New Zealand

**Keywords:** structural variants, single-nucleotide polymorphisms, *Chrysophrys auratus*, growth, feature selection, prediction

## Abstract

Background: Genetic diversity provides the basic substrate for evolution. Genetic variation consists of changes ranging from single base pairs (single-nucleotide polymorphisms, or SNPs) to larger-scale structural variants, such as inversions, deletions, and duplications. SNPs have long been used as the general currency for investigations into how genetic diversity fuels evolution. However, structural variants can affect more base pairs in the genome than SNPs and can be responsible for adaptive phenotypes due to their impact on linkage and recombination. In this study, we investigate the first steps needed to explore the genetic basis of an economically important growth trait in the marine teleost finfish *Chrysophrys auratus* using both SNP and structural variant data. Specifically, we use feature selection methods in machine learning to explore the relative predictive power of both types of genetic variants in explaining growth and discuss the feature selection results of the evaluated methods. Methods: SNP and structural variant callers were used to generate catalogues of variant data from 32 individual fish at ages 1 and 3 years. Three feature selection algorithms (ReliefF, Chi-square, and a mutual-information-based method) were used to reduce the dataset by selecting the most informative features. Following this selection process, the subset of variants was used as features to classify fish into small, medium, or large size categories using KNN, naïve Bayes, random forest, and logistic regression. The top-scoring features in each feature selection method were subsequently mapped to annotated genomic regions in the zebrafish genome, and a permutation test was conducted to see if the number of mapped regions was greater than when random sampling was applied. Results: Without feature selection, the prediction accuracies ranged from 0 to 0.5 for both structural variants and SNPs. Following feature selection, the prediction accuracy increased only slightly to between 0 and 0.65 for structural variants and between 0 and 0.75 for SNPs. The highest prediction accuracy for the logistic regression was achieved for age 3 fish using SNPs, although generally predictions for age 1 and 3 fish were very similar (ranging from 0–0.65 for both SNPs and structural variants). The Chi-square feature selection of SNP data was the only method that had a significantly higher number of matches to annotated genomic regions of zebrafish than would be explained by chance alone. Conclusions: Predicting a complex polygenic trait such as growth using data collected from a low number of individuals remains challenging. While we demonstrate that both SNPs and structural variants provide important information to help understand the genetic basis of phenotypic traits such as fish growth, the full complexities that exist within a genome cannot be easily captured by classical machine learning techniques. When using high-dimensional data, feature selection shows some increase in the prediction accuracy of classification models and provides the potential to identify unknown genomic correlates with growth. Our results show that both SNPs and structural variants significantly impact growth, and we therefore recommend that researchers interested in the genotype–phenotype map should strive to go beyond SNPs and incorporate structural variants in their studies as well. We discuss how our machine learning models can be further expanded to serve as a test bed to inform evolutionary studies and the applied management of species.

## 1. Introduction

Intraspecific genetic variation provides the substrate for hereditary evolutionary change [1]. Genetic variation has most commonly been assessed using molecular markers that quantify patterns defined by variation at one or a few base pairs, such as SNPs, AFLPs, and microsatellites. However, it is becoming increasingly recognized that structural variation represents a significant, yet often poorly understood, source of genetic variation [2,3]. It has been shown that structural variants, which are defined as genomic variants between individuals of the same species, can affect the position, order, direction, and composition of one or multiple nucleotide sequences and include chromosomal insertions, deletions, translocations, inversions, and duplications. It is only within the past 10–15 years, aided by the development of genomic technologies such as high-throughput 2nd-generation sequencing and later 3rd-generation sequencing, that the extent of intra- and interspecific structural variation has been investigated in a number of non-model species [4,5,6]. 

Major advances in the field of computational genomics combined with increasingly high-quality whole-genome data and assemblies for non-model species have led to a re-evaluation of what constitutes important genomic variation between individuals. Recent studies show that structural variation commonly affects a significant portion of the genome [7,8] and, indeed, that structural variants can impact more base pairs in the genome compared to SNPs e.g. [9]. Increasingly, studies also demonstrate that structural variants are key modulators of phenotype with significant consequences for fitness [3,10]. For example, in the African malaria vector *Anopheles gambiae,* inversion polymorphisms are linked to desiccation resistance [11], and in the balsam poplar *Populus balsamifera* L., copy number variations are linked with adaptive environmental variations linked to biotic and abiotic stress responses [12]. In particular, chromosomal inversions are a common mechanism by which multiple characters are inherited as a single locus, as is found in the common fruitfly *Drosophila melanogaster*, where continental replicates of populations show that large cosmopolitan inversions are involved in a parallel adaptive environmental responses [13]. 

Improving our understanding of the genomic variants that underpin complex phenotypic traits is particularly important for traits that have a polygenic architecture, which is the case for the predominant number of quantitative traits [14]. As postulated by the infinitesimal model, quantitative traits, such as growth, are typically caused by many genes of small to moderate effect [15]. The vast majority of studies to date that have investigated quantitative polygenic traits have used SNP variants, while larger variants in the genome have rarely featured in such studies. This is in large part because polymorphisms in SNP data can be easily quantified with high-throughput methods, while for structural variant analyses, the sequencing approaches are not so cost-effective, and the associated bioinformatic pipelines are less developed. Compiling information about structural variation across the genome is challenging, in part because of the large diversity of variants and the size spectrum that they cover. For example, the detection of exact inversion breakpoints can be challenging, as this would require a range of sequence read sizes (e.g., Illumina HiSeq 2500 with read sizes of 100–250 bp), including long reads (e.g., PacBio RS II sequencing with read sizes of about 10,000–15,000 base pairs), to cover the entire spectrum of inversion polymorphisms in many species. 

Here, we investigate the roles of SNPs and structural variant polymorphisms in explaining the quantitative trait growth in the marine teleost species the Australasian snapper, *Chrysophrys auratus* (hereafter referred to as snapper). Our approach outlines an initial framework of how such an exploration could be achieved to highlight the challenges and opportunities and ultimately to guide future research. The study species for this work is a species of significant commercial, recreational, and cultural significance in New Zealand, where it is referred to as tāmure by Māori. Plant and Food Research has a long-term research programme on this species to develop it for aquaculture using selective breeding. The key economic trait that is selected for is growth, to yield an elite strain with superior growth qualities, something that has been achieved for its sister species the red sea bream, *Pagrus major*, and the related gilthead sea bream, *Sparus auratus* [16]. Until now, genomic selection for growth has been based on SNP information alone. However, a recent study on this species demonstrated that the structural variation in deletions, duplications, and inversions contributes to as much as three times as many base pair variants in the genome when compared to SNPs [9]. To quantify the relative roles of SNPs versus structural genomic variation in predicting growth (small, medium, or large size categories), we (1) compared counts of SNPs and structural variants occurring in different window sizes and examined their ability to correctly assign a fish to a correct size class, (2) compared the size assignment accuracy to a model that included a feature-selection step to filter the most informative data, and (3) discuss the relative roles of SNPs and structural variants in determining growth in this species, the general implications of these findings to other species, and how our initial framework could be expanded in the future.

## 2. Materials and Methods

We used 32 fish for which we had individual whole-genome Novaseq data (150 bp paired-end, coverage 15×) and length data at age 1 and 3. The methods for the DNA extraction and processing from this cohort have been published elsewhere [9,17,18], but we briefly summarise the salient points below.

### 2.1. Study Population and Phenotypic Growth Measurements

Snapper cohorts from a three-generation pedigree were reared in tanks at the Nelson Finfish Facility with consistent feeding, light, water flow, aeration, and tank design [17,18]. For growth measurements, the fork length was measured as the distance from the nose to the fork of the tail. These measurements were made when the fish were between 436 and 487 days (“year 1”) and again when they were between 1045 and 1131 days (“year 3”). Due to mortality, not every fish was measured during year 1 and year 3, and the final dataset consisted of 32 fish for which all data were complete.

### 2.2. Genomic Data Filtering and Mapping

**Figure 1:**
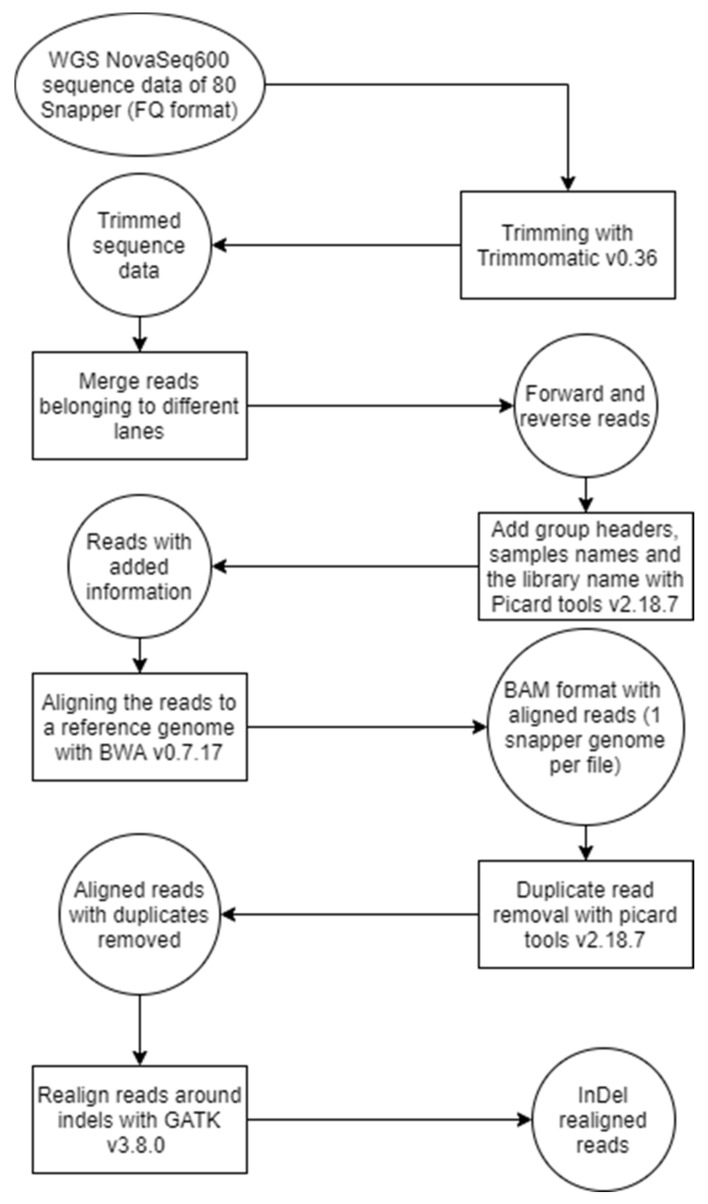
Overview of the processing of the genomic data.

NovaSeq600 S2 150 bp paired-end read data at an average coverage of 15× were generated for 80 snappers, including the 32 snappers used in this study (see Figure 1 for an overview of the genomic data preparation processes). The raw data of all 80 snappers were assessed for quality using FASTQC v0.11.7 [19], and reads were trimmed using Trimmomatic v0.36 [20], removing the first nine bases, trailing bases if their quality was less than 10, adapter sequences, and homo-polymer sequences. Reads with less than 75 bases after clipping were also removed. Trimmed reads were aligned to the snapper reference genome [9] using BWA v0.7.17 [21]. Duplicate reads were removed using Picard tools v2.18.7 [22]. GATK v3.8.0 [23], RealignerTargetCreator, and IndelRealigner were used to realign reads around indels. Bam files were filtered for reads with a minimum mapping quality (--min-MQ) of 20 using samtools.

#### Variant Calling

Small variants were called on all 80 snapper samples as a single cohort using FreeBayes v1.1.0 [24] with the following parameters: --report-genotype-likelihood-max, --min-base-quality 10, --min-mapping-quality 20, --genotype-qualities, --use-mapping-quality, --no-mnps, --no-complex, --max-complex-gap 50, --min-alternate-fraction 0.1, --min-repeat-entropy 1, --no-partial-observations--min-coverage 10, --max-coverage 500, and --pooled-continuous. Structural variants were called on individual fish using Parliament2 v2.0 [25], which consists of an ensemble of callers, including Breakdancer v1.4.3 [26], BreakSeq2 v2.2 [27], CNVnator v0.3.3 [28], Delly v0.7.2 [29], Lumpy v0.2.13 [30], and Manta v1.4.0 [31]. For variant frequencies and densities, structural variant call-sets of the full cohort of 80 fish and the 32 fish of the training and test sets were merged using SURVIVOR v1.0.3 [32].

To easily compare between single-nucleotide variant and structural variant data, SNP genotypes were summarised into counts occurring within 10 and 50 kb windows for each of the following genotypes: ‘.’, ‘0/0’, ‘0/1’, ‘1/1’, ‘0/2’, ‘1/2’, and ‘2/2’. Only variants with one or two alternative alleles were considered; variants with more than two alternative alleles were rare and were excluded from the analysis. For the development of features of structural variants, deletions, duplications, insertions, and inversions between 5 bp and 1 kb were counted within a 10 kb or a 50 kb window size. 

### 2.3. Model Construction, Testing, and Prediction

#### 2.3.1. Feature Selection Methods

Genotype data generated from whole-genome sequences typically contain very large numbers of variants and in turn, for use in machine learning, very large numbers of features (in this case, counts per genomic window). To reduce the features to those that contribute most to the prediction of size classes, we examined three feature selection methods, ReliefF, Chi-square, and mutual information methods. The ReliefF algorithm performs feature selection by scoring features based on nearest neighbour instance pairs, then selecting the top-scored features. If a feature has different values in the nearest neighbour pair but their classes (in this case, size classes) are the same, the score of this feature decreases. Conversely, if the feature values are different and their classes are different as well, then the score increases [33]. ReliefF is commonly used with high-dimensional genomic data. Furthermore, ReliefF implicitly considers interactions between features when performing feature scoring, which is very suitable for polygenic traits such as growth. We used the tuned ReliefF (TuRf) algorithm implemented in the scikit-rebate package [34], which uses a multi-round process where it eliminates the lowest-scored features iteratively until a predefined number of scores remain [34,35]. 

The Chi-square feature selection algorithm generates an estimated normal distribution of expected values of the different size classes and then tests the dependency between a feature and the class labels by placing it within this distribution. High dependency yields a high score, with the feature assessed as more useful [36]. Chi-square is a very commonly used statistical feature selector that usually produces relatively robust results. However, Chi-square does not consider interactions between features and might not work well for high-dimensional data. We used the SelectKBest implementation within scikit-learn [37,38].

The mutual-information-based feature selection method measures the shared information between a feature and the class labels or between two features. It achieves feature selection by removing features that have low mutual information with the class labels but high mutual information with other features and retaining those that have high mutual information with the class labels [39]. Mutual information was used because it is a commonly used method in genomics studies such as microarray studies [40] or gene expression studies [41], as those studies contain data of a high-dimensional feature space similar to this work. We used the mutual_info_classif implementation in scikit-learn [37,38]. 

#### 2.3.2. Classification Algorithms

We tested the prediction accuracy following feature selection on four common machine learning algorithms: random forest, Gaussian naïve Bayes, logistic regression, and K-neighbours. These were all performed with the default settings of scikit-learn v0.24.0 [37,38].

The random forest (RF) classification algorithm constructs an ensemble classifier containing a number of decision trees during the training process, each of which is a predictive model. RF combines the prediction of all the decision trees to produce a class label for each instance [42]. Logistic regression builds a function to map the input features to the probabilities of an instance belonging to a certain class [43]. K-nearest-neighbour (KNN), which is an instance-based learning (often called memory-based learning) method for classification using the distances between an instance (in the test/target set) and all the instances in the training set. The nearest neighbours vote for the class label of the target instance, and the Euclidian distance is commonly used as the distance measure in KNN [44]. Gaussian naïve Bayes (NB) is based on probability theory, which assumes features are conditionally independent of each other. NB calculates the (conditional) probability of each feature value for a class based on the training data and then predicts the probability of an unseen test instance belonging to each class [45]. 

#### 2.3.3. Model Building and Testing

**Figure 2:**
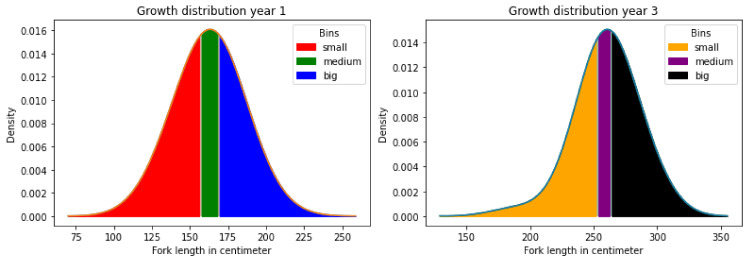
Density plot of the distribution of growth of 1- and 3-year-old snappers. The bins small, medium, and large are coloured red, green, and blue for year 1, respectively, and for year 3 they are orange, purple, and black, respectively.

Because the number of fish in the study was small, predicting a continuous variable (i.e., fish length) from count data (counts of the number of a type of variant in either a 10 kb or 50 kb window) was deemed impractical. Instead, we converted the continuous size measurement of length from tip to fork into three size classes (“small”, “medium”, and “large”) by separating the size measurements into 33.33% quantiles (see Figure 2). We also split the data into two datasets for the year 1 and year 3 age classes to enable a comparison of the prediction accuracies of the same fish at different time points.

For both the year 1 and year 3 datasets, we randomly split the data into 24 instances in the training set and 8 instances in the test set (Table 1). This distribution was maintained in the training and test sets for both the feature selection and the classification methods to prevent leakage. 

To compare the feature selection methods, we extracted the top features selected by TuRF, Chi-square, or mutual information. We then examined their subsequent prediction accuracy using the random forest, Gaussian naïve Bayes, logistic regression, and K-neighbours classification methods. We investigated how the feature number influences the prediction accuracy of growth by extracting the top 25, 50, 200, 250, and 500 ranked features and compared this to using all features (i.e., no feature selection). 

### 2.4. Result Annotation

To ask whether features were more likely to be associated with the presence of genes associated with growth within genomic windows, we extracted gene annotations from a gene annotation file described previously [46], and respective GO terms were extracted from a previously generated table [47]. The top 200 features were queried against the gene annotation file, and the number of hits was counted. We also tested the hypothesis that the features might be more likely to be associated with growth or development by counting any hits that had the terms “Growth” or “Development” in the cellular function annotation. We tested whether this was more likely than would be found through chance by using a permutation test that randomly sampled 200 features from the full feature set 1000 times. We counted the number of total hits or hits to “growth” or “development” in cellular function that the sampled features had to the annotation set and calculated the density distribution, including 99% confidence intervals.

Circos plots were generated using the R package circlize v0.4.1 [48] using bed files of variants merged from the 32 samples under study, which were filtered between 5 bp and 10 kb.

## 3. Results

### 3.1. Genetic Variant Catalogue

**Figure 3:**
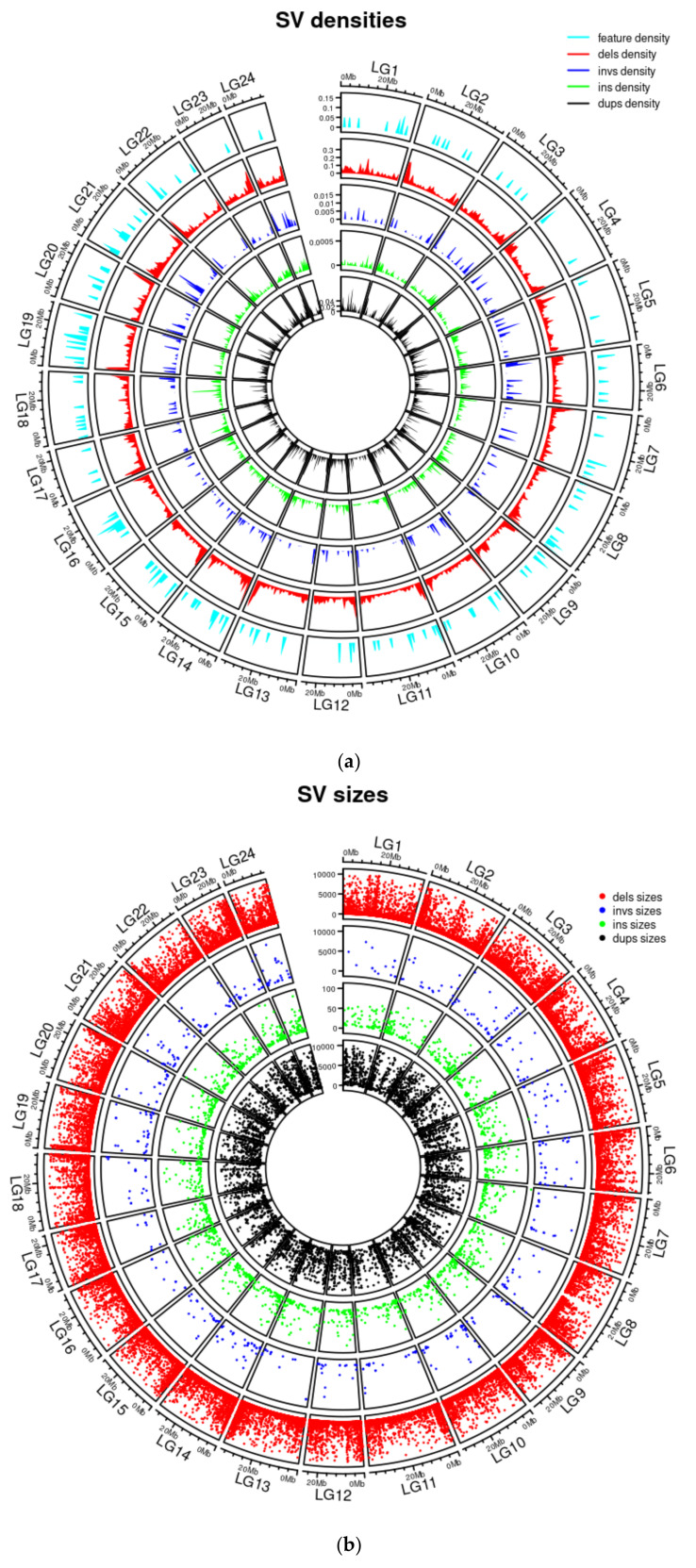
Circos plots of density (proportion of 100 kb genomic windows covered by variant class, (**a**) and locations and sizes (in base pairs, (**b**)) of structural variants called by Parliament2 within the 32 F2 samples and merged by SURVIVOR. In addition, the density plot shows the top 25 growth areas from the relief feature selection of SNPs and structural variants. In legends, dels = deletions, invs = inversions, ins = insertions, and dups = duplications.

A total of 21,573,217 small variants were called from all 80 snapper samples. The conversion of variants into features (counts of genotype categories within windows) resulted in a total of 102,648 features of 50 kb windows and 513,590 features of 10 kb windows. For the structural variants, there were 58,665 features of 50 kb windows and 293,493 features of 10 kb windows. When the structural variants between 5 bp and 50 kb were merged across the full cohort of 80 individuals, 10,789 duplications, 1092 inversions, 2225 insertions, and 78,719 deletions were counted, of which 6926 duplications, 825 inversions, 1731 insertions, and 63,017 deletions were counted within the 32 individuals under study for growth. Once filtered for variants between 5 bp and 1 kb, 1189 duplications, 294 inversions, 1731 insertions, and 43,946 deletions were counted. There were a total of 1,158,928 deletions, 511,009 insertions, 8204 inversions, and 33,290 duplications recorded. Figure 3a provides the densities of the structural variants between 5 bp and 1 kb alongside those of the 200 regions with the highest growth prediction with a 10 and 50 kilo base pair window on SNPs and structural variants. Figure 3b provides an overview of the locations and sizes of structural variants between 5 bp and 1 kb across the genome. 

### 3.2. Model Building and Testing

**Figure 4:**
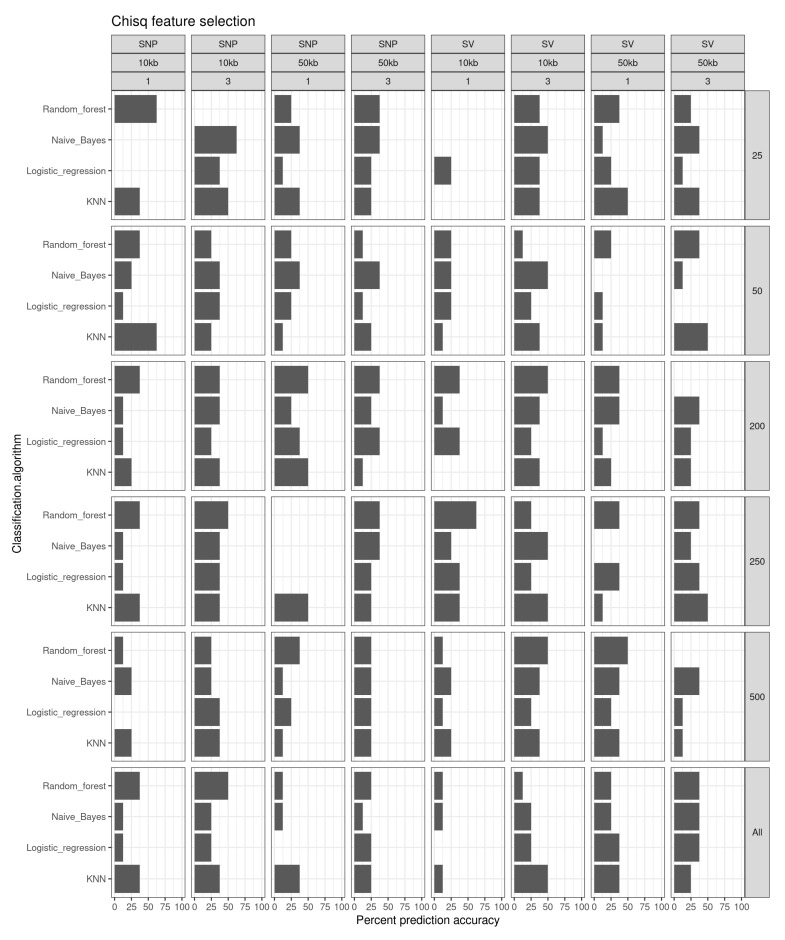
Prediction accuracy (%) for the different feature selection sets of classification algorithm using a Chi-square feature selection method.

Using no feature selection, the prediction accuracies to classify fish into the “small”, “medium” or “large” categories were generally low (e.g., Figure 4, “All” panel). All prediction probabilities were 50% or lower. The highest prediction probability was for the random forest and KNN classification methods (50% for each) for SNPs and structural variants, respectively, for 10 kb windows in 3-year-old fish.

Chi-square feature selection improved the prediction accuracies somewhat (Figure 4). The highest prediction accuracies (62.5%) were for the random forest algorithm in the top 25 SNPs in 10 kb windows using 1-year-old fish, the KNN classification on the top 50 SNPs in 10 kb windows in 1-year-old fish, naïve Bayes for the top 25 SNPs in fish at the age of 3, and random forest for the top 250 structural variants in the year 1 fish. All other combinations of features, classification algorithms, window sizes, and ages of fish had similar prediction accuracies to the no feature selection results.

**Figure 5:**
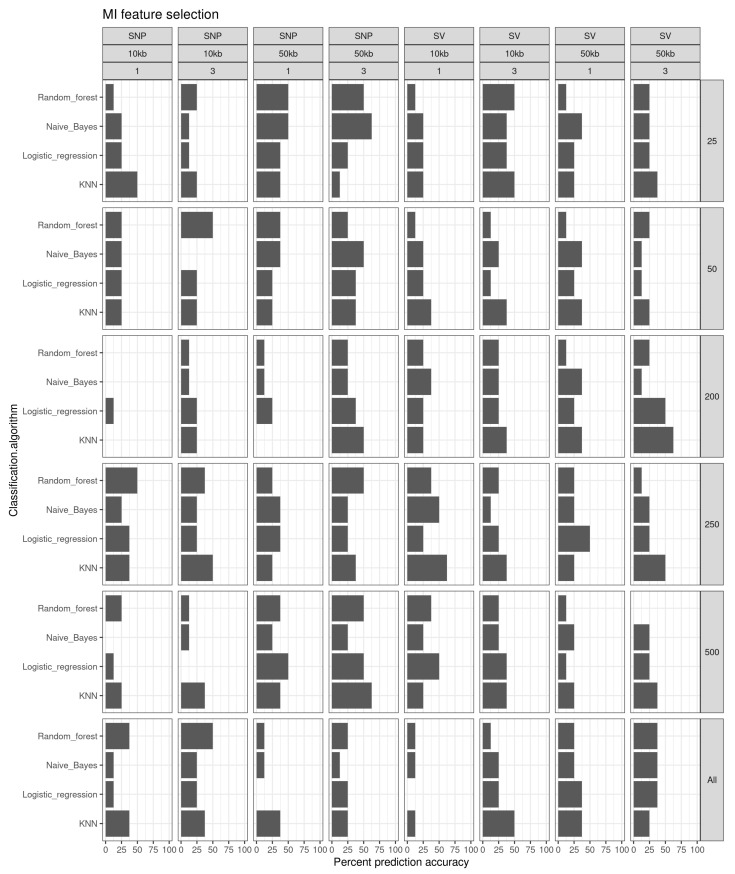
Prediction accuracy (%) for the different feature selection sets of classification algorithms using a mutual Information feature selection method.

The mutual information feature selection had top prediction accuracies of 62.5% using the KNN classification methods for 500 SNPs from 50 kb windows in year 3, 250 structural variants from 10 kb windows in year 1, and 200 structural variants from 50 kb windows in year 3 (Figure 5). In addition, naïve Bayes resulting in 62.5% prediction accuracies using the 25 top-scoring SNPs generated from 50 kb windows for year 3 fish. All other prediction accuracies were 50% or less for all other combinations of classification method, window size, fish age class, and feature number.

**Figure 6:**
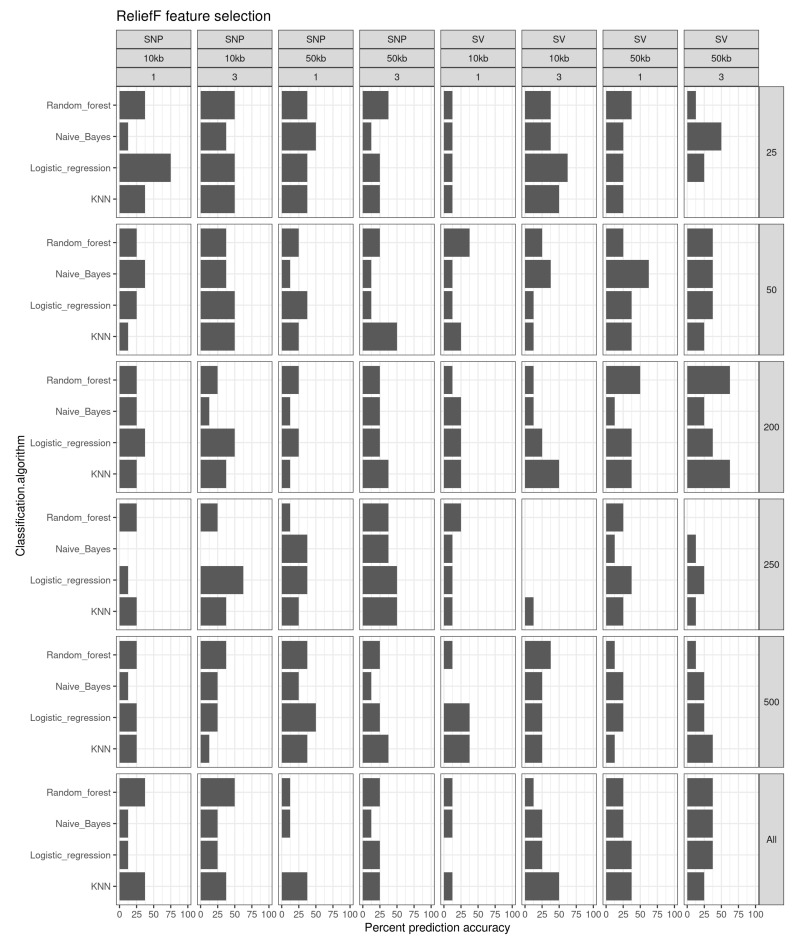
Prediction accuracy (%) for the different feature selection sets of classification algorithms using a ReliefF feature selection method.

ReliefF feature selection had prediction accuracies of 75% for logistic regression on SNPs generated from 10 kb windows (Figure 6). Logistic regression also generated prediction accuracies of 62.5% using the top 250 SNPs and 25 structural variants using 10 kb windows on year 3 fish. Other combinations of methods that yielded 62.5% prediction accuracies included naïve Bayes using the top 50 structural variants called from 50 kb windows on year 1 fish, random forest using the top 200 structural variants called from 50 kb windows on year 3 fish, and KNN using the top 200 structural variants from 50 kb windows in year 3 fish.

### 3.3. Annotation of Key Results

**Figure 7:**
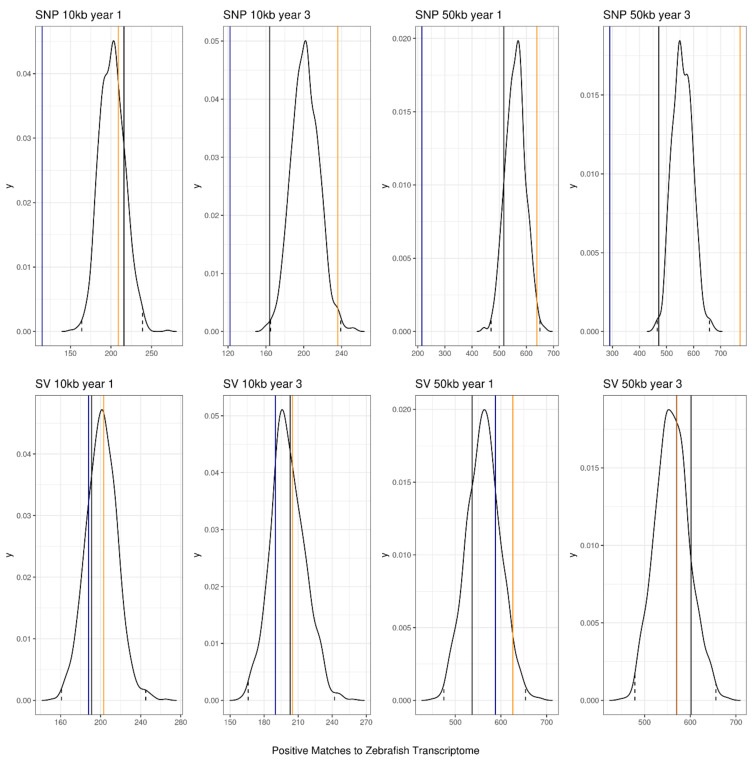
Permutation test of top 200 features compared to sampling 200 features randomly across the genomic windows. Solid lines are the number of positive hits to a genomic annotation of zebrafish (black is ReliefF feature selection, blue is mutual information feature selection, and orange is Chi-square feature selection). Dotted lines are the 99% confidence intervals for the 1000 sets of 200 randomly sampled features.

**Figure 8:**
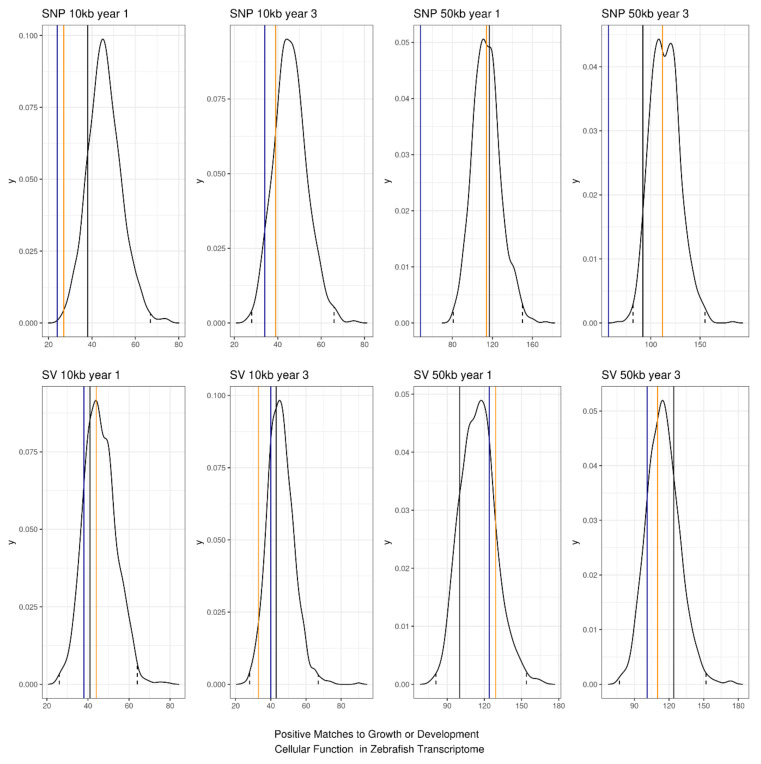
Permutation test of top 200 features compared to sampling 200 features randomly across the genomic windows. Solid lines are the number of positive hits to a genomic annotation of zebrafish that contain the words “Growth” or “Development” in the cellular function column (black is relief feature selection, blue is mutual information feature selection, and orange is Chi-square feature selection). Dotted lines are the 99% confidence intervals for the 1000 sets of 200 randomly sampled features.

Most features in the top 200 features were not significantly more likely to have a positive hit to the annotated zebrafish genome than would be found by chance alone (i.e., the counts fell below the 99% confidence interval in all but one case). The exception for this was for Chi-square feature selection using SNPs at 50 kb for years 1 and 3 and for SNPs called over 10 kb windows for year 3 fish (Figure 7). When this was filtered further to only include hits that related to ‘Growth’ or ‘Development’ in cellular function, there were no counts that were greater than the 95% or 99% confidence intervals (Figure 8). 

## 4. Discussion

How different types of genetic variation influence the phenotype is of key biological importance. The ease with which SNP data can be generated has led to significant progress in our understanding of how phenotypic trait variation is modulated by single changes in the base pair composition of the genome e.g. [47,49]. However, while structural variants impact large portions of the genome [2], there has been considerably less progress on how these underpin complex phenotypes. There is thus an urgent need to go beyond SNP variants if we want to better understand how the full extent of genomic variation impacts the phenotype and, ultimately, the eco-evolutionary trajectories of species [50,51,52]. 

Here, we explore the first steps towards understanding the predictive power of both SNPs and structural variant polymorphisms in explaining a complex quantitative growth trait by applying feature selection methods and machine learning models to our dataset. For our work, we chose the non-model species snapper (*Chrysophrys auratus*) because previous research on this species has generated significant genomic resources and an understanding of growth that we could leverage [17,18,47]. As such, our study explores some fundamental steps towards a fuller understanding of the genetic basis of growth and outlines some general considerations for future studies. Growth in vertebrates is a known polygenic trait with a complex genetic basis. Finding genomic regions associated with growth in snapper has been a significant endeavour until now, and detected regions have effect sizes of a few percent, something that is typical for highly polygenic traits [53]. Genomic growth regions are complex and often include regions that are inherited together, and epistasis and pleiotropy are common and epigenetic effects are being shown to be important in the regulation of gene activity. Thus, it is not just the genomic features that provide prediction signals to predict traits such as growth. It is also the environment of the fish and potentially their parents as well as the underlying architecture in the genome that are also potentially important features. We generated catalogues for SNPs and structural variants for 32 fish (2/3 were used as the training set, and 1/3 were used as the test set) to test different feature selection methods and machine learning models in predicting growth. To our knowledge, this is the first time that a study has explored the relative predictive power of these types of variants with these methods to explain a complex phenotypic trait such as growth. 

Overall, our approach to apply machine learning algorithms to genetic data, and to an extent the usual dataset of SNPs to include structural variants, has shown some promise, and we will outline the most important findings here. First, adding feature selection methods to retain the best-performing predictors for growth significantly improved model prediction accuracy, as predicted by previous theoretical and empirical work [54]. Without applying feature selection, the prediction accuracies were generally low (50% or less), indicating that this step is crucial to retain the genetic features needed by our model to make accurate predictions while discarding redundant features to reduce data dimensionality, remove noisy and irrelevant data, and thus preserve the most useful signals from the dataset. We also found that the prediction accuracy was slightly higher in year 3 than in year 1, regardless of the method applied (Figure 4, Figure 5 and Figure 6). This was possibly due to the more stochastic nature of growth during the first year of fish (e.g., stronger environmental impacts on larval and juvenile fish), while growth differences in older fish have a greater potential to reflect true accumulated genetic differences. 

Feature selection is a common method for high-dimensional genetic data [34,55], and other studies have found that ReliefF can perform quite well for genomic data, as an epistatic interaction can be included [56]. It is possible the poor performance of ReliefF in this work was due to the method of scoring of the SNP data. Rather than the presence or absence of an SNP, as is often scored, in this case we counted the numbers of SNPs for each genotype class across a window so that we could directly compare them with the counts of structural variants. Thus, we may have lost some of the associations between windowed regions that could occur. It was interesting, however, that the Chi-square feature selection did result in significantly more features that mapped to an annotated zebrafish genome for SNPs, particularly those scored across a 50 kb window in year 3 fish, with the number of hits to annotated regions falling well outside the 99% confidence intervals. However, it is likely that many of the other features do have some association with growth but that these have not been documented as such. Snapper is a non-model species, and relating regions of growth in this species to the more distantly related but well-annotated zebrafish genomes will always be an abstraction. Features that do map to the annotation may provide hypotheses for candidate growth regions of interest and as such could provide candidate regions for future exploration. These features might either be in gene regions associated with metabolic functions or they could be in linkage disequilibrium with coding regions, meaning some of the association might be correlative but not causal. 

Second, in some instances we were able to achieve prediction accuracies of 62% or higher for both the SNP and structural variant data. Thus, it does suggest that structural variants do provide some useful information that associate with the complex growth trait in this snapper population. This has not always been found to be the case. For example, in a study of 35,000 Holstein and Jersey cattle (*Bos taurus*), only a small proportion of total phenotypic variance was accounted for by structural variants [57]. It should be noted, however, that in this case structural variants were imputed from SNP data, which would have likely limited the range of detection and thus underestimated the likely predictive power. However, evidence is accumulating from various studies with wide taxonomic spread that structural variants are significant determinants of phenotypic traits and are associated with their regulation. A study on 1141 American lobsters (*Homarus americanus*) from 21 sampling sites revealed that copy number variants accounted for a higher genetic differentiation than SNP markers and were significantly associated with the annual variance in sea surface temperature, providing a strong empirical case that structural variants contribute to local adaptation [58]. Likewise, in tomato (*Solanum lycopersicum*), structural variants detected through long-read nanopore sequencing in 100 individual genomes were shown to be responsible for not just the difference in allele dosage but also a range of expression of phenotypes [59]. In some cases, just a few major structural variants are major determinants of adaptive traits and fitness, as seen in honeybees (*Apis mellifera),* where chromosomal inversions have been linked to local adaption [60], or sunflowers where major haploblocks are responsible for ecotypic differentiation [61]. We therefore think that it is likely that further examination of structural variants in species with diverse ecologies will document additional evidence in support for their significant contribution to phenotypic traits and fitness. This is particularly likely for species such as snapper, where the bp affected by structural variants outnumber those impacted by SNPs threefold and where many of these variants intersect with genes [46]. 

Third, in addition to the architectural complexity of genomic data, the cost and time investment for genome-wide scans in non-model species such as snapper commonly results in a low n (sample size), high p (parameter number) problem. This problem is described by the fact that it is generally more cost and time effective to screen for a large number of variants within an individual than it is to screen large numbers of individuals [62] as is common in the fields of, e.g., neuroimaging, genomics, motion tracking, eye tracking, and many other technology-based data collection methods that have led to a torrent of high-dimensional datasets. This is a well-known area where classical machine learning algorithms do not perform well [63,64]. However, despite small sample sizes being common and the fact that limited data are problematic for pattern recognition, only a limited number of papers have systematically investigated how the machine learning validation process should be designed to help avoid optimistic performance estimates. In our situation, we had 24 fish in the training set and predicted on 8 fish in the test set, and low numbers prevented the more traditional n-fold cross-validation procedures that precede testing. Indeed, it is also possible that our test and training sets did indeed have some leakage: related individuals were necessarily in both sets, which would have overinflated the test accuracies. Some degree of relatedness is also a typical find when screening individuals from the same sampling populations, particularly when movement dynamics are low. In addition, we have only screened a few of the more common machine learning algorithms. Undoubtedly, other untried algorithms will be found that perform better. For example, neural networks and other deep learning methods have been proposed as useful algorithms for genomic data, especially those with small sample sizes [64,65]. In addition, it may be that combining SNPs and structural variant datasets can provide a more complete view of the complexity of the genome, potentially explaining growth variation in a more complete manner. 

In conclusion, our work outlines a promising approach to finding genetic variants associated with complex traits and showcases how one can go beyond SNP data to also account for structural variant data. While our results indicate that SNPs can account for some of the variation in phenotypic traits, our results also highlight that structural variants should not be overlooked and that they carry significant additional phenotype information. Therefore, we recommend that researchers interested in the genotype–phenotype map should strive to go beyond SNPs in their work to capture the underpinnings of quantitative traits, such as growth. This will allow researchers to harness more of the available catalogue of genetic variants in the genome that are ultimately impacting the phenotype. In addition, future efforts need to increase the sample size of fish to better account for the genetic variation underlying this complex trait and to minimise to some extent the large n, small p issue in many biological datasets used for machine learning approaches. 

## Figures and Tables

**Table 1:** The number of fish in the small, medium, and large categories for the year 1 and year 3 fish in the training and test datasets.

	Year 1 Fish		Year 3 Fish	
	Training Dataset	Test Dataset	Training Dataset	Test Dataset
Small	10	1	9	0
Medium	4	6	4	2
Large	10	1	10	6

## Data Availability

As the genomic data of this species are from a taonga and thus culturally important species in Aotearoa New Zealand, the data have been deposited in a managed repository that controls access. Raw and analyzed data are available through the Genomics Aotearoa data repository at https://repo.data.nesi.org.nz/. This was done to recognise Māori as important partners in science and innovation and as inter-generational guardians of significant natural resources and indigenous knowledge.
